# Ruptured Baker’s Cyst: A Diagnostic Dilemma

**DOI:** 10.7759/cureus.18501

**Published:** 2021-10-05

**Authors:** Kuldeep Bansal, Anuj Gupta

**Affiliations:** 1 Spine Surgery, Indian Spinal Injuries Center, Delhi, IND; 2 Orthopedics & Spine, Triveni Ortho & Spine Center, Delhi, IND; 3 Spine, Max Superspeciality Hospital, Delhi, IND

**Keywords:** baker's cyst, popliteal, ruptured baker's cyst, conservative, deep vein thrombosis (dvt)

## Abstract

A ruptured Baker’s cyst is a rare presentation and may mimic deep vein thrombosis (DVT) or acute thrombophlebitis. In rare cases, it may present with infection or compartment syndrome. We present our experience related to a case of a ruptured Baker’s cyst and its management. A 54-year-old female presented to us with knee pain, which was initially managed conservatively. After six weeks, she came to us with severe pain and swelling in her left calf and foot. It was an acute presentation and DVT was suspected initially. Ultrasound color Doppler showed no DVT and then MRI revealed it to be a ruptured Baker’s cyst. The patient was subsequently managed conservatively and her condition improved in 12 weeks of follow-up. A high index of suspicion and knowledge is required to diagnose a ruptured Baker’s cyst, and most of the patients respond well to conservative management.

## Introduction

Baker’s cyst, also known as a popliteal cyst, is formed due to fluid accumulation in the pre-existing bursae at popliteal fossa [1]. The most common complaint of patients with this condition is a palpable swelling at the popliteal area with an associated vague pain. But in very rare cases, a Baker’s cyst can rupture and can have a presentation similar to that of deep vein thrombosis (DVT) or acute thrombophlebitis [2,3]. The ruptured Baker’s cyst can also present with infection and, rarely, compartment syndrome [4].

In this report, we present a case of a ruptured Baker’s cyst and discuss its clinical presentation, diagnosis, and our experience in managing the patient.

## Case presentation

A 54-year-old female presented to us with a complaint of left knee pain for six months. On examination, the knee range of motion was full, with no ligamentous laxity. There was tenderness at the femoral attachment of the lateral collateral ligament (LCL) and also at pes anserinus. Also, a Baker’s cyst was present at the popliteal fossa. Local infiltration with an injection of bupivacaine and methylprednisolone acetate was done, and the patient was educated about quadriceps- and hamstring-strengthening exercises. The patient reported good relief after the procedure.

After 1.5 months, she presented with severe swelling in the left lower limb extending from knee to foot. There was no history of any trauma and no signs of infection. On physical examination, swelling could be appreciated in the left leg and foot. The swelling was more prominent at the dorsal aspect of the leg. There was tenderness at the middle one-third of the calf. The dorsiflexion of the foot seemed to intensify the pain (Homans sign). The plantarflexion power of the foot was normal. Hence, we suspected DVT and got an ultrasound color Doppler of the patient. The color doppler showed no signs of DVT.

**Figure 1:**
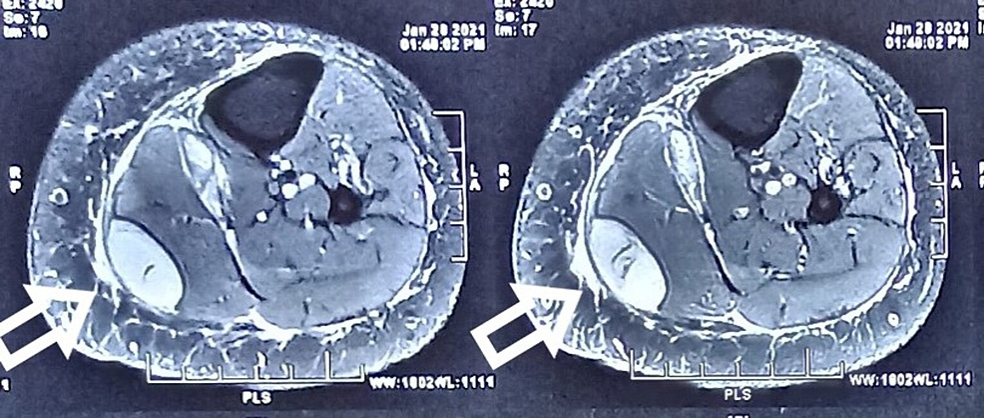
Axial T2-weighted image showing hyperintense cyst (arrows)

**Figure 2:**
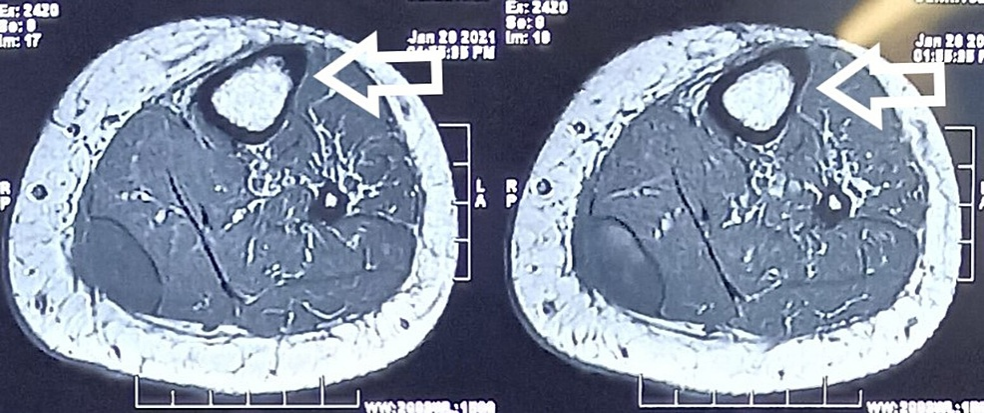
Axial T1-weighted image showing hypointense cyst (arrows)

**Figure 3:**
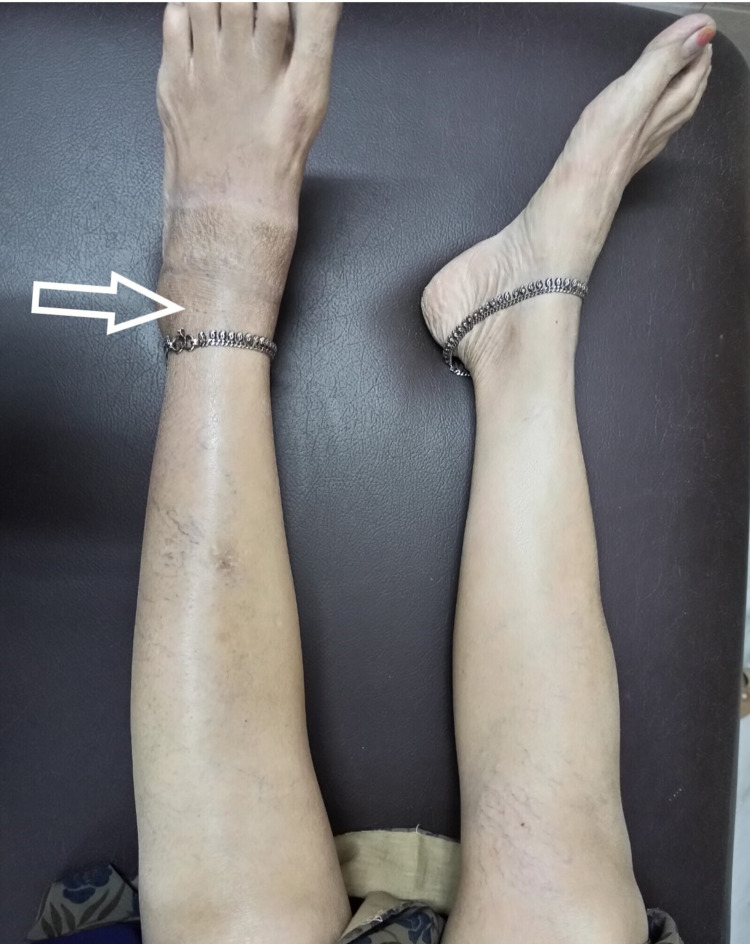
Clinical picture showing swelling on left foot (arrow)

**Figure 4:**
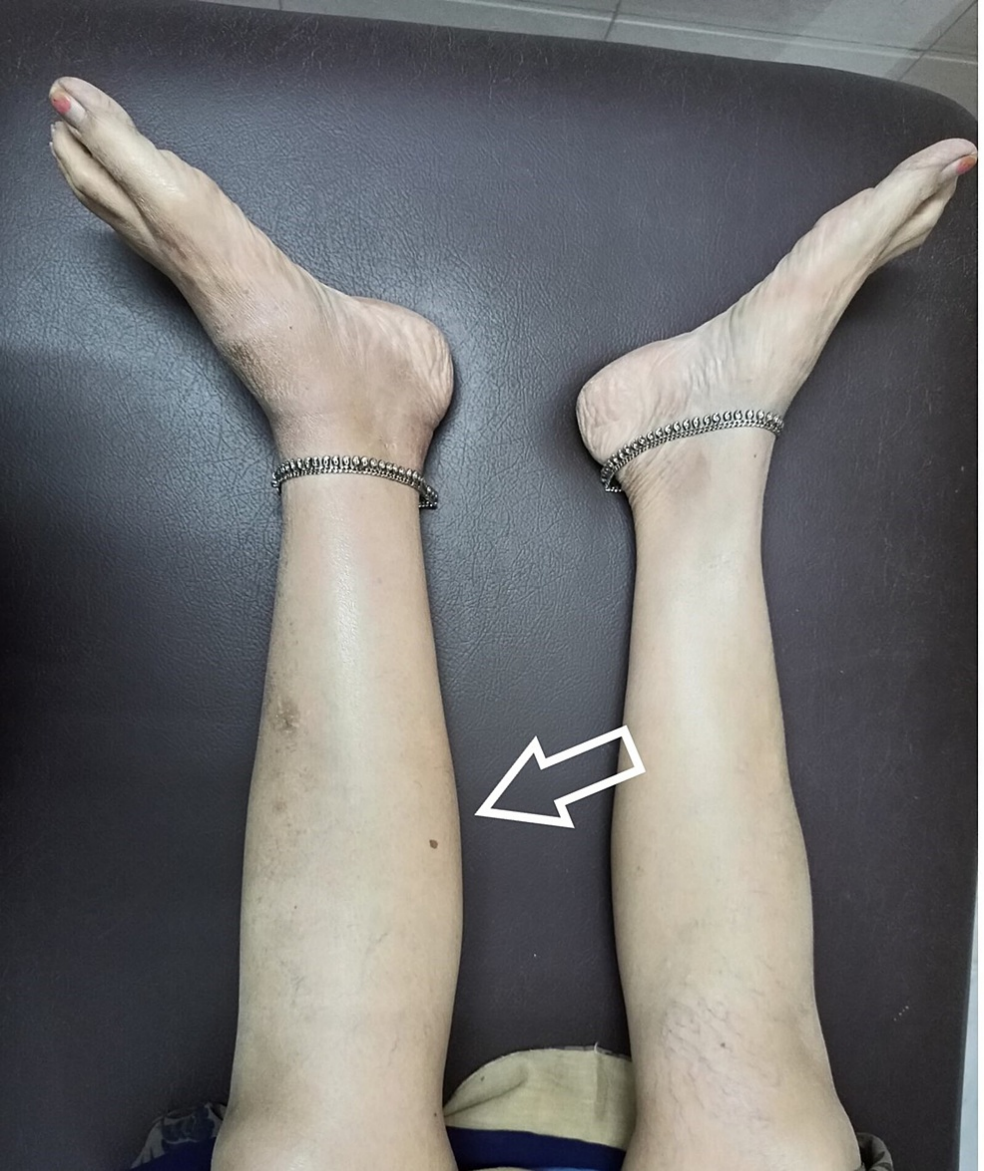
Clinical picture showing swelling on the left leg (arrow)

Then, on careful questioning, she gave a history of pain exacerbation while doing vigorous exercises of quadriceps and hamstring. Also, she stated that she had noticed some fluid going down the leg. This prompted us to suspect a ruptured Baker’s cyst, and an MRI of the leg was performed. The MRI showed a fluid collection at the intermuscular plane, which correlated with the site of tenderness at the calf (Figure 1, 2, 3, 4).

The patient was managed conservatively and kept on regular follow-up. On subsequent follow-ups at two weeks, six weeks, and 12 weeks, the swelling completely subsided and the patient became pain-free.

## Discussion

Baker’s cyst is one of the most common cystic lesions of the knee joint. It is formed by the distension of gastrocnemio-semimembranosus bursa. Unlike other bursae of the knee joint, this bursa communicates with the cavity of the knee joint [1]. The incidence of Baker’s cyst ranges from 5 to 38% [5] and it increases with age since the integrity of the joint capsule decreases with age. Most cases of Baker’s cyst do not require any active intervention, but in rare cases, it can rupture and may require urgent attention. The cause of rupture is not well defined in the literature but there are a few obvious reasons like direct trauma and iatrogenic causes. In our patient, it possibly occurred due to trauma while she was doing exercise.

The presentations in cases of ruptured Baker’s cyst closely resemble those of DVT [2]. It could present with swelling and redness in the calf region. Also, the Homans sign, which causes pain in the calf region on dorsiflexion of the foot, can be seen. Moreover, due to inflammation at the calf, there will be pain while squeezing the calf, which indirectly points towards DVT. That is why a ruptured Baker’s cyst is also termed pseudothrombophlebitis [6]. Another presentation closely resembling that of ruptured Baker’s cyst is acute thrombophlebitis [3]. Our patient also presented with similar complaints seen in DVT and acute thrombophlebitis, which initially misguided us, and we got the diagnosis confirmed with ultrasound color Doppler. Another common presentation in ruptured Baker’s cyst is ecchymosis at the popliteal region [7]. Though not seen in our patient, it has been described as an important sign of this condition. One more sign described in the literature related to ruptured Baker’s cyst is the crescent sign [8]. Bruising seen below the ankle is known as a crescent sign. It signifies fluid in the calf gravitating towards the ankle. This sign is difficult to elicit on physical examination [9], but only clinical signs are described in this condition.

Ultrasound is the initial investigation of choice as it rules out DVT and also indicates the possibility of a ruptured Baker’s cyst. The collection of fluid in the intermuscular plane points to a ruptured Baker’s cyst. Although a few authors consider ultrasound as the key diagnostic tool [8], in our experience, ultrasound could not diagnose the condition. In our experience, MRI can diagnose the condition better than ultrasound.

The ruptured Baker’s cyst usually presents with acute pain and swelling. Hence, the only treatment required is pain management with anti-inflammatory medications and local heat [10]. Surgical intervention is indicated only in a few cases, in which drainage of the collection is required [7]. Our patient improved with anti-inflammatory medications and other conservative management. The swelling subsided gradually and the pain decreased to a great extent in 6-12 weeks. There are cases reported in the literature wherein patients presented with compartment syndrome [4]. In such cases, prompt diagnosis and intervention can salvage the limb of the patient.

## Conclusions

The ruptured Baker’s cyst is a rare entity and is often missed on initial presentation as its symptoms closely resemble those of DVT or acute thrombophlebitis. Though most of the patients respond well to conservative management, a high index of suspicion is needed to make an early diagnosis.
